# First Large Scale Application with Self-Healing Concrete in Belgium: Analysis of the Laboratory Control Tests

**DOI:** 10.3390/ma13040997

**Published:** 2020-02-23

**Authors:** Tim Van Mullem, Elke Gruyaert, Robby Caspeele, Nele De Belie

**Affiliations:** 1 Magnel-Vandepitte Laboratory, Department of Structural Engineering and Building Materials, Faculty of Engineering and Architecture, Ghent University, Tech Lane Ghent Science Park, Campus A, Technologiepark Zwijnaarde 60, B-9052 Gent, Belgium; Tim.VanMullem@UGent.be (T.V.M.); Robby.Caspeele@UGent.be (R.C.); 2 Department of Civil Engineering, KU Leuven, Ghent Technology Campus, Gebroeders De Smetstraat 1, 9000 Ghent, Belgium; Elke.Gruyaert@kuleuven.be

**Keywords:** Bacterial self-healing concrete, large-scale demonstrator, site trials, permeability tests

## Abstract

Due to the negative impact of construction processes on the environment and a decrease in investments, there is a need for concrete structures to operate longer while maintaining their high performance. Self-healing concrete has the ability to heal itself when it is cracked, thereby protecting the interior matrix as well as the reinforcement steel, resulting in an increased service life. Most research has focused on mortar specimens at lab-scale. Yet, to demonstrate the feasibility of applying self-healing concrete in practice, demonstrators of large-scale applications are necessary. A roof slab of an inspection pit was cast with bacterial self-healing concrete and is now in normal operation. As a bacterial additive to the concrete, a mixture called MUC+, made out of a Mixed Ureolytic Culture together with anaerobic granular bacteria, was added to the concrete during mixing. This article reports on the tests carried out on laboratory control specimens made from the same concrete batch, as well as the findings of an inspection of the roof slab under operating conditions. Lab tests showed that cracks at the bottom of specimens and subjected to wet/dry cycles had the best visual crack closure. Additionally, the sealing efficiency of cracked specimens submersed for 27 weeks in water, measured by means of a water permeability setup, was at least equal to 90%, with an efficiency of at least 98.5% for the largest part of the specimens. An inspection of the roof slab showed no signs of cracking, yet favorable conditions for healing were observed. So, despite the high healing potential that was recorded during lab experiments, an assessment under real-life conditions was not yet possible.

## 1. Introduction

Concrete is a widely used material for buildings and infrastructure. It is relatively cheap and behaves well under compressive stresses, but cracks easily under tensile stresses. Consequently, cracks are a common sight in many concrete structures. These cracks are, amongst others, a result of restrained shrinkage, mechanical loading, and thermal expansion. In most cases, the presence of cracks does not directly threaten the load bearing capacity of structures due to the presence of tensile reinforcement which takes over the stresses of the cracked concrete in the tensile zone. Yet, the presence of cracks can impede (part of) the function of a structure, such as liquid tightness in e.g., (underground) parking garages, tunnels, locks, and canals, Additionally, cracks are an aesthetic disruption and they reduce the durability and, as a consequence, the service life. At the location of cracks, the steel reinforcement is no longer protected by a concrete cover. As a result the load bearing capacity is indirectly threatened due to harmful substances, such as water, oxygen, carbon dioxide, chlorides, and others, which can easily migrate to the reinforcement and cause corrosion. In order to prevent this, repair actions have to be carried out. However, these are expensive, time-consuming and often difficult to execute due to accessibility problems, aside from causing large economical losses due to downtime and e.g., traffic jams in the case of infrastructure applications. A study of 10 years ago indicated that Europe spent about half of its construction budget on repairing structures [1]. This is not surprising, as a significant amount of bridges were constructed in the 1950s and 1960s [2]. More recent data showed that Belgium invested only 0.3% of its gross domestic product of 2016 in road infrastructure, out of which less than half went to maintenance [3]. These investments have been decreasing over time and this highlights the need for structures to operate longer and with less maintenance and repair interventions. An increased service life of structures is also an important way to combat global warming. Using data from 2009, it was estimated in a recent study that the global construction sector had a share of 23% in the global CO_2_ emissions [4]. Constructing infrastructure and buildings which could operate longer at a high performance would have a significant impact on the environment.

A promising new material to reduce or even prevent the influence of cracks on the degradation of concrete and thereby increasing the service life, is self-healing concrete. This type of concrete contains specially developed additives so that it can close cracks by itself. Without external intervention, the material is able to regain its original liquid tightness and/or strength. Several types of self-healing concrete have already been developed [5] and different test procedures and modelling approaches to investigate the self-healing capacity have been proposed [6,7]. Up until now, most research is focused on small lab scale mortar specimens, due to the high cost of the self-healing additives. It can be expected that when these materials are produced in bulk their cost will decrease. Several laboratory studies have already investigated large-scale elements with dimensions comparable to those commonly found in practice. Van Tittelboom et al. [8] cast beams (25 cm × 15 cm × 300 cm) with the addition of encapsulated polyurethane or with the addition of superabsorbent polymers. They were cracked in a four-point bending setup and a comparison of the self-healing efficiency was done. The same size of beams and a similar test approach was used to investigate the crack filling in concrete with ureolytic mixed self-protected bacterial cultures [9]. Araújo et al. [10] cast beams (40 cm × 20 cm × 250 cm) with concrete containing mixed-in glass capsules or polymethyl methacrylate capsules (both filled with water repellent agent) to validate the self-sealing efficiency of these two encapsulation methods. Gruyaert et al. [11] report non-destructive tests and corrosion monitoring on beams with the same dimension which were cast with concrete with the addition of either a Mixed Ureolytic Culture (MUC) or superabsorbent polymers. In another study of the effect of various shear span to effective depth ratios on the self-healing behavior of engineered cementitious composites (ECC), beams were tested with a cross-section of 12.5 cm × 25 cm and a length varying between 113 and 242 cm [12].

It should be noted that the previously mentioned studies on large-scale elements were still executed in a lab environment. The final goal of any self-healing technique should be to demonstrate its self-healing potential in an in-situ real life structure. Currently this brings some challenges with regards to the upscaling of the production of the self-healing additives. Other important aspects are the size of the demonstrator element, its accessibility, and the expected exposure conditions. After all, many healing techniques require contact to liquid water [13,14,15,16] or at least a very humid environment [17], with the exception of some encapsulated liquid polymers which are able to harden upon contact with air, another polymer component, or moisture which is present in the concrete matrix [8,18].

Due to the combination of these challenges, application of self-healing in the field is rather limited, though a few examples can be found. Dry [19] placed brittle tubes near the surface of four full-scale bridge decks. As the bridge deck was shrinking, the tubes ruptured and created controlled expansive joints. Tubes were also placed in the bulk of the bridge decks where they were able to fill shear cracks. In another application, bacterial self-healing concrete was used to make an irrigation canal in Ecuador liquid tight [20,21]. Alkaliphilic bacteria and calcium lactate were impregnated in light-weight aggregates. These aggregates, together with indigenous fibers to control the crack formation, were added to a local concrete mix to cast a three meter long section of concrete lining the canal. An evaluation of the self-healing capacity was not possible, since the concrete did not crack. In the UK self-healing concrete panels were constructed next to a highway [22,23]. They were loaded in a controlled way and they underwent the same weather cycles as the adjacent highway. Several self-healing techniques were tested in these panels: microcapsules containing mineral healing agent, bacteria infused into lightweight perlite aggregate particles with a flow network to provide nutrients, shape memory polymers, and a vascular flow network providing a mineral healing agent. It was found that the different self-healing techniques achieve their best potential under different applications. Therefore, knowledge of the damage phenomenon is necessary for choosing the correct self-healing technique and achieving a good self-healing. Al-Tabbaa et al. [24] reported that the panel with the microcapsules was able to heal cracks faster and more complete than a control panel, resulting in a significant impermeability recovery. Aside from the application of self-healing additives in self-healing concrete, several case studies with self-healing repair products have also been reported [9,20,21,25].

The current paper reports on the first application of self-healing concrete in Belgium. The traditional concrete in a roof slab of an underground inspection chamber was replaced by bacterial self-healing concrete. Due to the function of the structure, the bottom of the roof slab is always accessible for inspection.

The bacteria in the self-healing concrete were provided in a powder form called MUC^+^, which is a combination of a granular Mixed Ureolytic Culcture (MUC) and anaerobic granular bacteria. Additionally, a urea and a calcium source were also added to the concrete to provide the bacteria with a food source. Previous results for mortar indicated that a good regain in liquid tightness could be obtained [26]. At the same time that the roof slab was cast, accompanying lab specimens from the same concrete batch were cast. A small preliminary analysis has already been reported [27,28]. This paper discusses all the different tests which were executed at the lab and elaborates on the behavior of the self-healing roof slab.

## 2. Trial Site

Antwerp is the largest city of Belgium and is located on the right bank of the river Scheldt. Daily there are a lot of traffic problems. This is a consequence of the fact that the motorway ring road around Antwerp is not complete and has insufficient capacity. To remediate this problem the Oosterweel link will be constructed as from 2020. This ambitious project will close the ring road, as well as modernize part of the existing road infrastructure. To close the ring road, a third tunnel will be constructed to pass the river Scheldt, in addition to the two already existing tunnels—the Kennedy tunnel and the Liefkenshoek tunnel [29].

**Figure 1:**
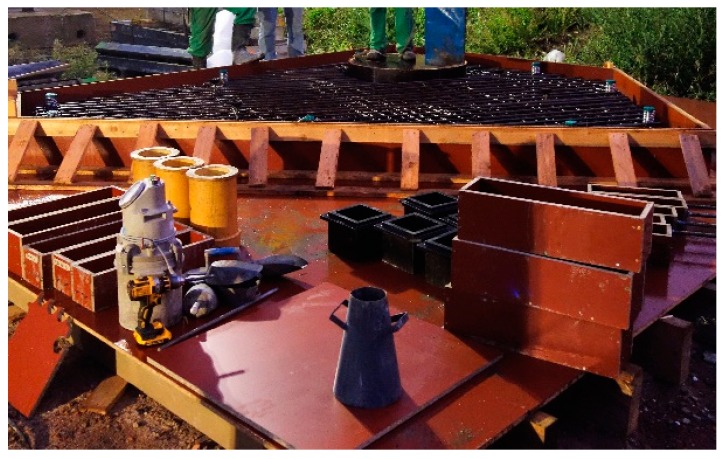
Formwork of the roof slab with the molds of the accompanying lab specimens in front.

**Figure 2:**
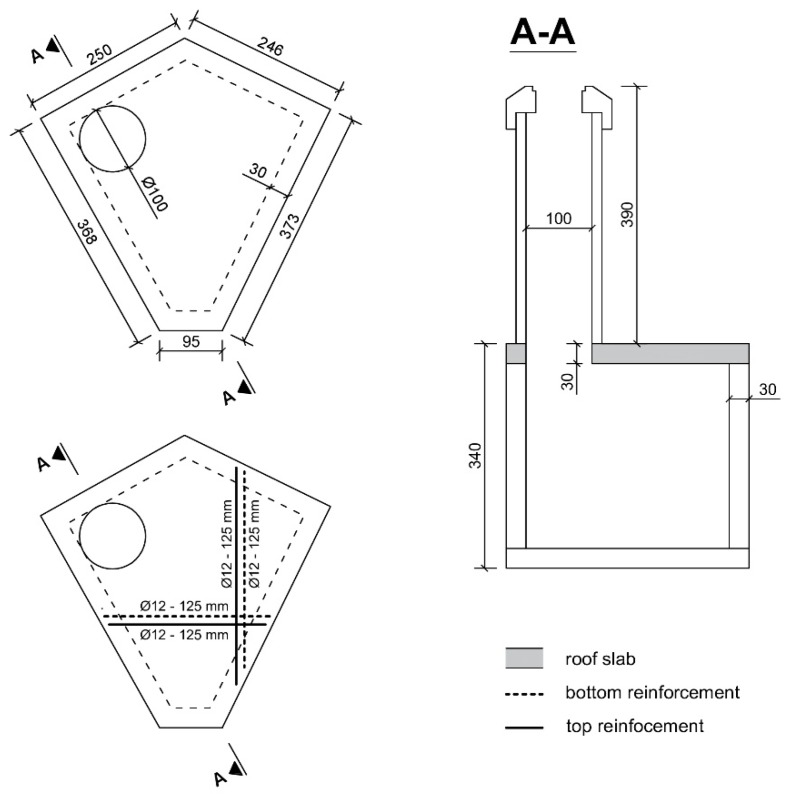
Top view with dimensions of the roof slab (top left). Top view with principle top and bottom reinforcement of the roof slab (bottom left). Side view of the inspection chamber with the roof slab highlighted (dimensions are in mm).

To allow for a smooth execution of the substantial project, preparatory works have been undertaken. Part of these preparatory works were the relocation of drainage pipes from the company Aquafin, which is responsible for water treatment in Belgium. The construction project on which the trial site was chosen was executed by a consortium of different contractors, called THV Schijnpoort. Different trials sites to test the efficiency of self-healing bacterial concrete in a real life application were considered. In the end, a roof slab of an inspection chamber of one of the drainage pipes was chosen as the most preferred trial site. It was expected that there would be liquid water, or at least a very high humidity, in the inspection chamber, which is a prerequisite for healing [6]. Furthermore, the roof slab would be located 3.9 m below ground level but would remain accessible through a manhole. This way the bottom of the roof slab, where cracks are expected to appear, will remain accessible over time for inspection of crack formation and subsequent crack healing. It is noted that self-healing concrete can have many applications, but not all elements are easily and safely accessible throughout their lifetime without thorough preparation (e.g., bridge girders which need an aerial work platform to be accessible or tunnels for which traffic has to be diverted for safety reasons). The roof slab was cast on site next to the building pit. Figure 1 shows the formwork of the roof slab, as well as the molds of the accompanying lab specimens (see also Section 3.3). Figure 2 shows two schematic top views of the roof slab, one with the dimensions of the slab and one with the principle reinforcement. Additionally, Figure 2 also shows a side view of the inspection chamber in which the roof slab has been highlighted. To allow for access through the manhole via a precast shaft, there was a circular opening with a diameter of 100 cm in the roof slab. The principle top and bottom reinforcement consisted of nets with bars with a diameter of 12 mm and a spacing of 125 mm. The concrete cover was 50 mm. After curing, the slab was positioned on top of the inspection chamber using a crane (see also Section 5). The thickness of the walls on which the roof slab rests are equal to 30 cm, see Figure 2.

Due to the fact that the inspection pit had to be fully operational throughout the classic design life, it was not possible to load the roof slab by a controlled loading configuration. All the loads on the roof slab were the result of real life loads during construction and operation.

In operating conditions, there is a bicycle lane above the roof slab. The roof slab was designed taking into account the traffic load from the bicycle lane, the weight of the ground layer above the roof slab and an axis load of a truck parked on the manhole. The concrete design was done taking into account possible corrosion due to carbonation, attack due to freeze/thaw, and chemical attack.

## 3. Materials and Methods

### 3.1. Bacterial Healing Agent

The used bacterial healing agent was MUC^+^, which was developed and produced by the company Avecom in the EU-FP7 project HEALCON [30]. This MUC^+^ consists out of a Mixed Ureolytic Culture (MUC) and anaerobic granular bacteria. MUC is a non-axenic bacterial culture with a capacity to sporulate. It is comparable to a Cyclic EnRiched Ureolytic Powder (CERUP) described in previous work [31]. As microbial source for production a side-stream of a vegetable treatment factory was used. To stimulate the growth of the desired bacteria a high urea content was added together with the feed and thermo cycles were induced to act as a stress factor and to stimulate sporulation. Upon activation the spores germinate and precipitate calcium carbonate which allows for a closure of cracks. The advantages of this granular MUC product are that there is no need for encapsulation [11], and compared to axenic cultures the risk of contamination, as well as the operational expenditure (OPEX) cost, is far lower [31]. Anaerobic granular bacteria were added to the MUC to improve the overall efficiency of the bacterial agent, due to the high CO_2_ production and CaCO_3_ precipitation capacity of these granular bacteria. The resulting MUC^+^ is a granular product (comparable to sand) which can be added dry to a concrete mix.

To promote the activity of the MUC^+^, urea and calcium nitrate tetrahydrate were added as nutrients and as a calcium source.

### 3.2. Concrete Composition and Casting

**Figure 3:**
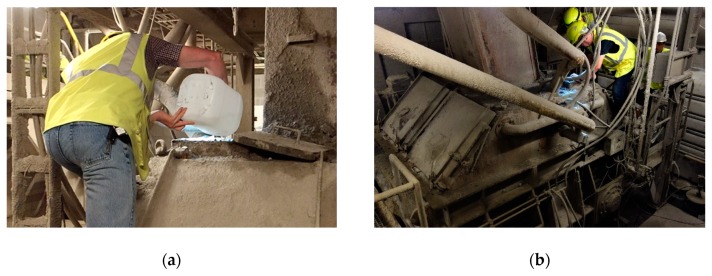
(**a**,**b**) Addition of bacterial agent, urea and calcium nitrate tetrahydrate through inspection hatch above industrial concrete mixer.

A batch of 3.5 m³ of concrete for casting of the roof slab and the accompanying lab specimens (see also Section 3.3) was mixed in a concrete plant near the trial site. The mix proportions are given in Table 1. As a slump keeper (superplasticizer A containing a polycarboxylic ether), respectively a water reducer (superplasticizer B, containing a lignosulfonate), two superplasticizers were added to the mix. The dosage of the bacterial healing agent, the urea and calcium nitrate tetrahydrate was for each component separately 1 mass % relative to the cement weight, see Table 1. The urea and calcium nitrate tetrahydrate were dissolved in part of the mixing water in order to promote a uniform distribution of these agents throughout the concrete. The bacterial agent was not dissolved in water, because it was produced in a dry powder form. Several options on what would be the best method to add the self-healing additives to the mixing process were explored. In the end the bacterial agent, the urea and the calcium nitrate tetrahydrate were added separately to the mix by means of an inspection opening at the top of the mixer, see Figure 3.

The concrete was transported to the construction site by a mixing truck (Antwepren, Belgium). Prior to casting, the concrete was shortly mixed in the truck. At the construction site the slump (determined according to NBN EN 12350-2) amounted to 90 mm and the air content (determined according to NBN EN 12350-7) was 4.5%. This slightly increased air content can partly be explained by the longer mixing as a result of the manual addition of the bacterial healing agent and the nutrients.

### 3.3. Laboratory Control Tests

Casting of the slab and of the lab specimens happened simultaneously. Both were compacted using a vibrating needle (THV Schijnpoort, Antwerp, Belgium). After casting, both the roof slab and the lab specimens were covered by a plastic foil. The lab specimens were kept next to the roof slab for 5 days after which they were moved to the lab. They were demolded at an age of 6 days and were stored in a curing room (20 °C, > 90% RH). To test the strength of the concrete, cubes (150 mm × 150 mm × 150 mm) were cast. Cylinders (h = 300 mm, Ø = 150 mm) were cast to determine the modulus of elasticity. Additionally, laboratory control specimens were cast to investigate the visual crack healing, the water permeability, and the capillary water absorption.

#### 3.3.1. Visual Crack Healing

**Figure 4:**
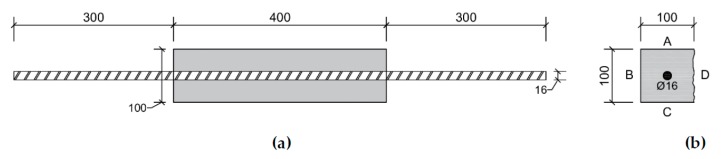
(**a**) Side view of reinforcement in prismatic specimen used to evaluate visual crack healing; (**b**) Cross-section of prismatic specimen used to evaluate visual crack healing (A: top surface, B: side surface, C: bottom surface, and D trowelled surface) (dimensions in mm).

**Figure 5:**
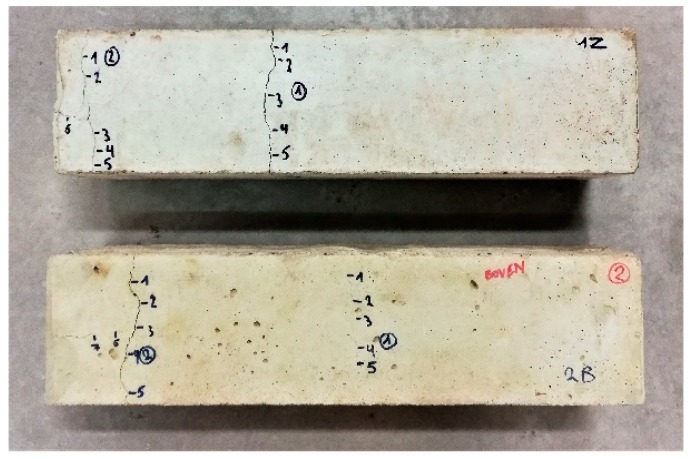
Specimens for visual crack healing with an indication of measurement locations. The reinforcement bar running along the length of the specimens has already been sawn off.

Prismatic specimens (100 mm × 100 mm × 400 mm) were cast with one reinforcement bar (Ø 16 mm) with a length of 1000 mm, see Figure 4. The reinforcement bar was centrally positioned, so that 300 mm was sticking out of each side of the specimens. In total 4 specimens were cast. At an age of 5 weeks the specimens were cracked in a uniaxial tensile test similar to the cracking of prismatic mortar specimens by Wang et al. [16]. The reinforcement bar was clamped at both ends in a tensile setup (Amsler 100, SZDU 230,). At a displacement of 0.01 mm/s a tensile load was applied on the bar allowing it to deform in a plastic way. After cracking of the specimens, the parts of the reinforcement bars sticking out of the specimens near the end surfaces were sawn off and these end faces were coated. Due to the tensile load on the reinforcement bar two cracks were formed, one of which was in the center of the specimen and one was a combination of a perpendicular and a longitudinal crack near the end of the specimens, see Figure 5. One specimen had an additional crack near the end.

After cracking of the specimens, the crack width was measured at several locations along the crack by taking pictures with a camera positioned on an optical microscope (DFC295 camera mounted on Leica S8APO microscope). The locations were chosen at random, yet all locations were representative of their respective crack and care was taken that they were well distributed along the length of the cracks. As an example, Figure 5 shows a picture (taken with a normal digital camera) of 2 specimens on which an indication is given of the locations (indicated by a blue marker) where the cracks were measured with a microscope, see also Section 4.2. The reported crack width for a location is the average of measurements in 4 to 5 specific points in the picture.

After characterization of the crack widths, the specimens were healed: two specimens were stored in wet/dry cycles (4 h wet/2 h dry) using tap water (20 °C, 60% RH during the dry period), one specimen was submerged in tap water (20 °C), and one specimen was stored in a moist environment (20 °C, 95% RH). After 3 and 6 weeks, the specimens were temporarily removed from their healing condition to evaluate the crack closure by re-measuring the crack width in exactly the same locations. Care was taken that the orientation of the specimens stayed the same, i.e., the same side was always positioned at the bottom during the healing condition, see Figure 4b.

#### 3.3.2. Capillary Water Absorption

**Figure 6:**
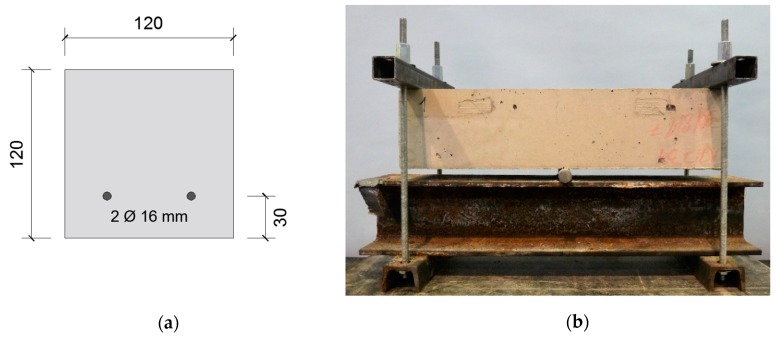
(**a**) Cross-section of specimen used for capillary water absorption (dimensions in mm). (**b**) Manual three-point bending setup to crack the capillary water absorption specimens.

To test the capillary water absorption, 4 prismatic specimens (120 mm × 120 mm × 500 mm) were cast with 2 reinforcement bars (Ø 6 mm) located at 30 mm from the bottom side of the specimens, see Figure 6a. The prisms were cracked at an age of 6 weeks in a manual three-point bending setup, see Figure 6b. The bottom side of the specimens was facing up. The two upper supports had a width of 3 cm. The distance between the two upper supports was 44 cm. The bar which forms the third support had a diameter of 2 cm. The specimens were loaded until the crack width after unloading was wider than 300 µm (the maximal allowable crack width for a wide variety of concrete classes prescribed by EN 1992-1-1:2004 [32]. The crack width was measured in minimally 6 locations according to the approach described in Section 3.3.1. One specimen had crack branching (i.e., parallel cracks) over more than half of its width. The crack width in this branching zone was determined as the sum of the crack width of both parallel cracks, as this gave a similar crack width to the zone without crack branching. It is noted that this approach is not entirely correct, yet the reported crack widths are only indicative and no direct link between the crack width and the capillary water absorption is made. One of the four specimens remained uncracked. This specimen was sawn in three prisms of 120 mm × 120 mm × 150 mm. These prisms were used to measure the capillary water absorption on uncracked concrete.

All specimens were coated with two layers of a two-component epoxy. A zone with a width of 40 mm centered on the crack (or the middle of the specimens for the uncracked prisms) remained uncoated. Aside from this zone the complete bottom side of the specimens was coated, as well as the lower 20 mm of the sides (with exception of the uncoated zone with a width of 40 mm).

The cracked specimens were placed for 10 days in a climate room at a temperature of 33 °C. The uncracked blocks were placed in the climate room 4 days later, since they would dry out quicker due to their smaller volume. In the last 24 h in the climate room the mass loss was smaller than 0.035% (unconventionally, one sample had a mass increase of 0.030%). The moisture content of all specimens was also measured using a TQC Concrete Moisture Meter 24h before the specimens were taken out of the climate room, as well as at the moment the specimens were taken out of the climate room. The use of this device allowed the determination of the moisture content by measuring the electrical impedance. It was noted that 24 h of extra drying did not change the moisture content; furthermore, the moisture content of the uncracked blocks and the cracked prisms was nearly identical. This allowed to conclude that both had dried to approximately the same degree.

An initial capillary water absorption measurement was then done by placing all the specimens in one container on spacers. The water level was 4 to 5 mm above the bottom of the specimens. After 5 min, 15 min, 30 min, 60 min, 90 min, 2 h, 3 h, 4 h, 6 h, 8 h, and 24 h the specimens were taken out and their weight was measured after removing water drops on the surface with a wet cloth.

After the initial capillary water absorption test, the specimens were saturated in tap water for 27 weeks. After this healing period, the crack width was measured again. The specimens were subsequently dried. The duration of the drying period of the uncracked blocks was again shorter. They were stored in a humidity chamber (20 °C, >90% RH) prior to drying. When the weight and the moisture content was comparable to the weight before the initial capillary water absorption test, a final capillary water absorption test was executed.

The sealing efficiency $SE_{abs.}$ of a specimen can be calculated according to Equation (1):

(1) $$SE_{abs.}=\frac{SC_{crack,unh.}-SC_{crack,heal.}}{SC_{crack,unh.}-{\bar{SC}}_{uncr.,heal.}}\cdot 100\%$$

where $SC_{crack,unh.}$ is the sorption coefficient of a cracked specimen prior to healing, $SC_{crack,heal.}$ is the sorption coefficient of the same cracked specimen after healing, and ${\bar{SC}}_{uncr.,heal.}$ is the average sorption coefficient of the healed uncracked specimens. The sorption coefficient is the slope of the linear regression curve of the water uptake (in g) with respect to the square root of time (in √h).

#### 3.3.3. Water Permeability

**Figure 7:**
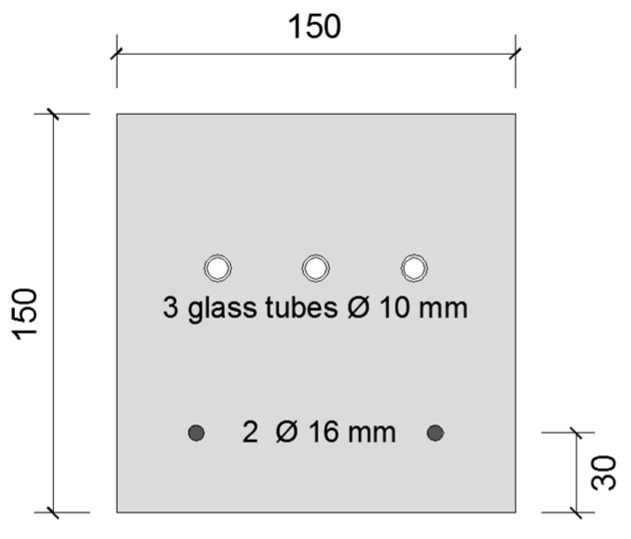
Cross-section of water permeability specimen (dimensions in mm).

Five prismatic specimens (150 mm × 150 mm × 550 mm) were cast with 2 reinforcement bars (Ø 6 mm) located at 30 mm from the bottom side of the specimens, see Figure 7. The specimens also had 3 glass tubes (Ø_out_ = 10 mm, Ø_in_ = 8 mm) running over their entire length which were located 92 ± 2 mm from the bottom side of the specimens, similar as in the work of Feiteira [33]. At an age of 6 weeks the specimens were cracked in a similar way as the capillary water absorption specimens (see Section 3.3.2). The distance between the upper supports was equal to 43 cm. During cracking of the beams it was possible to hear the racking of the glass tubes.

The width of the crack on the bottom side of the specimens was determined in a similar way as described in Section 3.3.2. The reported mean crack widths are an average of minimally 6 measurements at 6 different locations along each crack.

**Figure 8:**
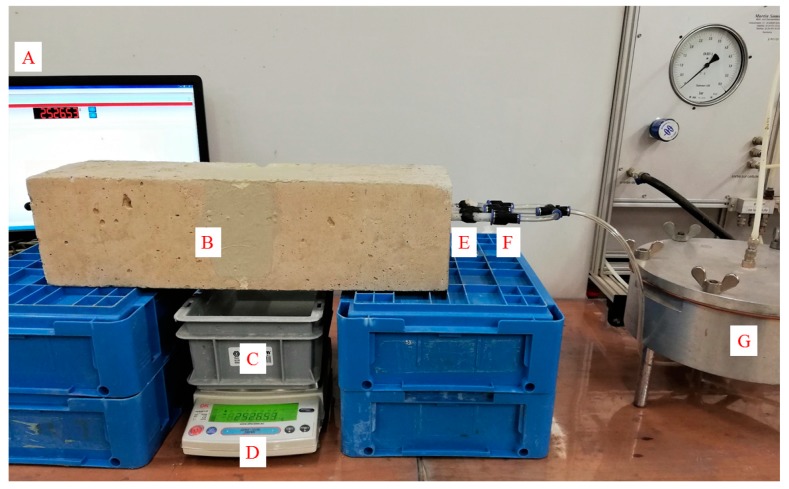
Water permeability setup (A: computer recording the weight measured by the scale, B: epoxy to seal the lateral side of the specimen, C: container to capture the water leaking out of the crack from the bottom side of the specimen, D: scale, E: glass tubes which run along the entire length of the specimen, F: connection of the glass tubes to a pressurized water reservoir via flexible tubing, and G: pressurized reservoir).

Prior to executing an initial permeability test, the specimens were submersed in tap water for 5 days. This was done to prevent water uptake of the matrix during the test. After this saturation period, the glass tubes on one side of the specimens were connected to a water reservoir pressurized at 1 bar, see Figure 8. The other side of the glass tubes was sealed. Due to the applied pressure, water from the reservoir flowed into the glass tubes and from there into the cracks. The mass of water leaking out of the bottom of the specimens was continuously recorded for 15 min. To prevent leaking from the lateral sides of the specimens, the cracks at the lateral sides of the specimens were sealed with an epoxy after cracking. If leakage from the lateral sides would be allowed, this could give too conservative a value [34]. After all, these prismatic specimens are used to analyze the healing behavior of a roof slab, in which there can be no lateral water leakages.

After the initial permeability test, the specimens were saturated in tap water. The permeability test was repeated every 3 weeks, up until 27 weeks healing in tap water. Out of the permeability tests, it was possible to calculate the sealing efficiency $SE_{perm.}$ of a specimen after a certain time t:

(2) $$SE_{perm.}=\frac{q_{initial}-q_{t}}{q_{initial}}\cdot 100\%$$

with $q_{initial}$ the flow rate determined from the initial permeability test and $q_{t}$ the flow rate determined form a permeability test after a time *t*.

## 4. Results and Discussion of Laboratory Control Tests

### 4.1. Compressive Strength and Modulus of Elasticity

The compressive strength at an age of 7, 28, and 93 days was determined according to NBN EN 12390-3 on concrete cubes with a side length of 150 mm. The concrete strength was determined both at the lab and in the mixing plant. The results are given in Table 2. The values are the average of 2 specimens. It is noted that there is still a significant strength increase between 28 and 93 days. This can partly be explained by the high slag content of the cement.

The modulus of elasticity was determined on 3 cylinders of diameter 150 mm and height 300 mm based on NBN B 15-203. It was equal to 31.8 GPa.

### 4.2. Visual Crack Healing

**Figure 9:**
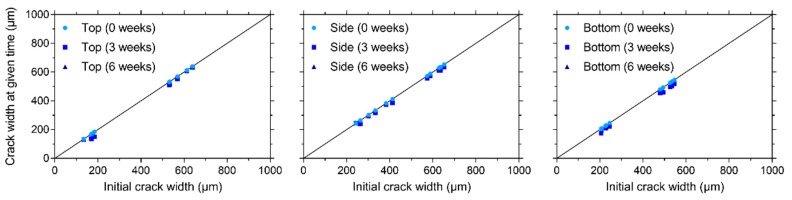
Evolution of the crack width under > 90% RH on the top, side, and bottom surfaces.

**Figure 10:**
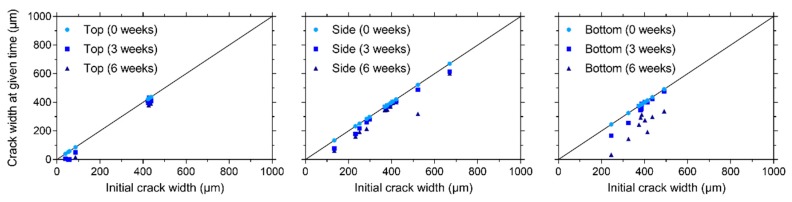
Evolution of the crack width under saturation on the top, side, and bottom surfaces.

**Figure 11:**
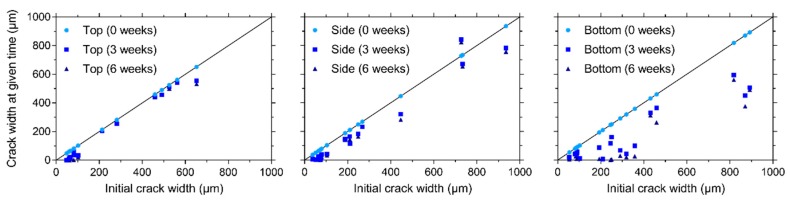
Evolution of the crack width under wet-dry (W/D) cycles on the top, side, and bottom surfaces.

After cracking of the prismatic specimens for visual crack healing, the crack width of all cracks at different faces (except the trowelled face) of the specimens (top, one of the sides, and bottom, see Figure 4b) was measured. Subsequently, specimens were stored in different healing conditions (95% RH, saturation, and wet-dry (W/D) cycles) and the crack width was measured after 3 and 6 weeks of healing. The orientation of the specimens was always kept the same, e.g., the top face was always oriented upwards during healing. Figure 9, Figure 10, and Figure 11 give the evolution of the crack width for the different faces of the specimen after storage in, respectively, >90% RH, saturation and wet-dry (W/D) cycles. In general, it can be seen that there is quite a bit of variation on the initial crack width: it varies from about 37 µm to 935 µm. Note that some specimens had even higher crack widths at some locations (>1000 µm), yet these are not displayed in the figures.

Regardless of the face of the specimen, the crack closure is very limited for the specimen stored at 95% RH, see Figure 9. The maximum relative crack closure is: 17.0%, 8.4%, and 13.9% for the top, side, and bottom face, respectively. It should be noted that the five locations with the lowest crack width were located at the top (134–185 µm). In contrast, the minimum crack width at the side and bottom was, respectively, 243 µm and 206 µm. This could explain why the highest relative crack closure could be found for a location at the top face of the specimen.

The healing of the cracks is clearly better for the specimen subjected to water submersion, see Figure 10. Three locations at the top of the specimen with a mean crack width varying between 52 and 58 µm obtain a perfect crack closure. The maximum crack closure for the cracks on the side of this specimen is 53.9% for a location with a mean crack width equal to 134 µm. The maximum crack closure for the bottom of the specimen is significantly higher—86.3% for a location with a mean crack width of 245 µm. From these results, it is difficult to conclude which face of the specimen gives the best crack closure for a submersed healing condition. The top face obtains a higher maximum crack closure, yet it should be noted that this is obtained for cracks with a mean width smaller than 100 µm, while the location with the smallest mean crack width for the bottom face is 245 µm. In any case, it appears that the cracks at the bottom have a more consistent crack closure even at higher mean crack widths.

On all faces of the specimens subjected to W/D cycles, a maximum relative crack closure of 100% was recorded, see Figure 11. The maximum mean crack widths for which a closure of 100% was measured were: 61 µm (top), 72 µm (side), and 245 µm (bottom). Additionally, several locations obtained a nearly perfect crack closure (>90%): five locations at the top (with a crack width varying between 48 µm and 81 µm), two locations at the side (with a crack width varying between 72 µm and 76 µm), and nine locations at the bottom (with a crack width varying between 54 µm and 358 µm). From these results, along with the global visual impression of Figure 11, it is clear that the bottom side obtains the best crack closure. There are two possible causes for this. The first being the effect of gravity, where calcium carbonate which is produced by the bacteria and which is not well bonded to the crack walls falls down and is deposited in a lower part of the crack. The second cause is related to the movement of the water. From Figure 9, it can be concluded that the bacteria need water to form calcium carbonate. When calcium carbonate is formed and the wet cycle is at an end, the water can take the calcium carbonate down in the crack where it is again deposited. Since the bottom face of the specimen also gave the most consistent crack closure for permanent saturation (Figure 10), in which there is no water flow, the first cause related to the influence of gravity is more plausible.

Comparing the results of the different exposure conditions, it is clear that the best results are obtained for W/D cycles and the worst results are obtained for a moist environment (>90% RH). Likewise, Luo et al. [14] found that the amount of crack closure for mortar with alkali-resistant bacteria was negligible in moist conditions (25 °C, 90% RH). Wang et al. [16] tested mortar with *Bacillus sphaericus* spores encapsulated in hydrogels and they also found that liquid water needs to be provided to ensure a good bacterial activity. The better healing for the W/D cycles, compared to the submersed healing condition, can be explained by the presence of atmospheric CO_2_ during the dry cycles which does not need to dissolve in water but is readily available at the crack which is slowly drying. This CO_2_ is able to react with portlandite to form calcium carbonate [35,36,37,38,39], aside from the calcium carbonate produced by the bacteria.

The most important cracks which will form on the large roof slab will be a result of the loads applied during operating conditions. These cracks will be mainly located on the bottom surface. The results described in this section indicate that these cracks would have a high healing potential, as long as liquid water would be present.

**Figure 12:**
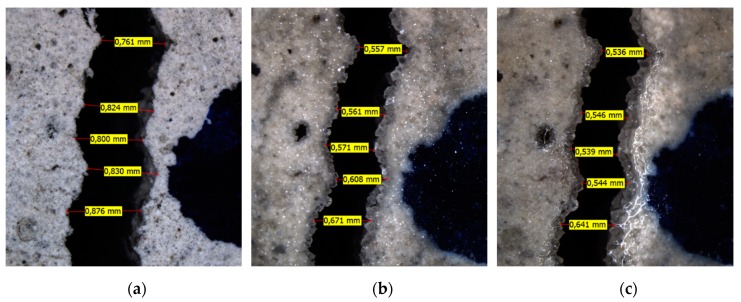
Crack location on the bottom faces of a specimen subjected to W/D cycles (**a**: initial, **b**: 3 weeks of healing, and **c**: 6 weeks of healing).

Figure 12 gives an overview of the healing of a relatively wide crack location located on the bottom of a specimen subjected to W/D cycles. The formation of crystals in the crack is evident. Though it also appears that part of the crack closure is not the result of healing products, but rather an elastic closure. This is possibly the result of a relaxation in the steel.

**Figure 13:**
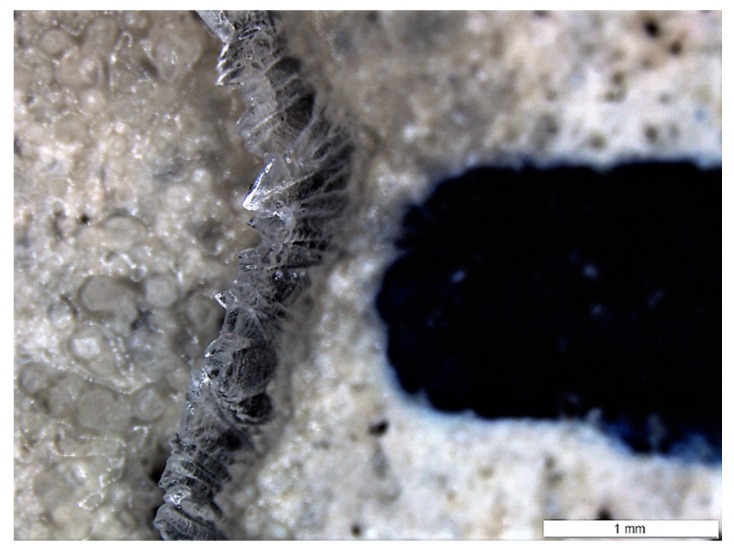
Formation of stalactites on a crack location on the bottom face of a specimen subjected to W/D cycles.

It is stressed that the evaluation of the crack width after a certain amount of healing is not straightforward. Often it becomes challenging to identify the same points in different pictures due to changes on the surface of the concrete and the deposition of healing products. Especially if the healing products are not deposited inside the crack. As an example, Figure 13 shows healing products which have formed a stalactite on one of the crack walls. The measurement of the crack width of such locations is subjected to a high degree of subjectivity.

### 4.3. Capillary Water Absorption

**Figure 14:**
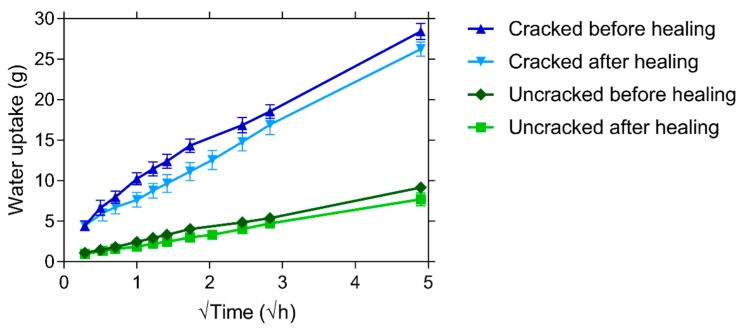
Mean cumulative water uptake in function of the square root of time of both cracked and uncracked specimens before and after healing. The length of the error bars represent two times the standard deviation.

The mean cumulative mass increase due to capillary water uptake in function of the square root of time for both the cracked and uncracked specimens before and after healing is given in Figure 14. The healing period causes only a slight decrease in the mean water uptake of the uncracked specimens. This indicates that there is only a small densification of the concrete matrix. Similarly, there is also only a limited decrease in the capillary water uptake of the cracked specimens after healing. The mean water uptake in the first 5 min is even equal before and after healing, which could potentially be explained by small differences in the water height during the two tests. Table 3 gives the sorption coefficients before and after healing, out of which it is possible to calculate a sealing efficiency using Equation (1). The sealing efficiency varies between 0.9% and 15.3%. This limited improvement in capillary water uptake is somewhat surprising. Visual evaluation of the cracks before and after healing showed that the mean crack width decreased by 63%, see Table 3. It should be indicated that the crack width after healing was determined on the same day that the specimens came out of saturation. The subsequent drying period will have resulted in a slight decrease of the autogenous healing, due to a physical shrinkage of the hydrated cement paste, and thus a slight increase of the crack width [35]. Additionally, it is noted that the mean crack width is determined from measurements of the crack width at discrete points, see Section 3.3.2. So, although the mean crack width has decreased, there can be regions along the crack where there is only a very limited precipitation of healing products and these regions can act as a highway for the ingress of water into the crack. They are often areas where the crack width is wider, e.g., due to a loss of an aggregate near the crack wall, and as a results a perfect closure of the cracks near the surface can be improbable, see also Section 4.2.

A partial crack closure does not necessarily result in a significant drop of the capillary water uptake as there are two counteracting phenomena. As the tortuous crack walls come closer together, the mass of the water in the crack will decrease. Yet, this is counterbalanced by an increase of the capillary action due to which water will ingress quicker in the crack from where it will migrate into the adjacent matrix. Up until now, there is no consensus on the tipping point in capillary water absorption tests of cracked cementitious materials. The closure of the cracks near the surface is also not a guarantee for the deposit of healing material on the crack walls in the interior of the specimens.

### 4.4. Water Permeability

**Figure 15:**
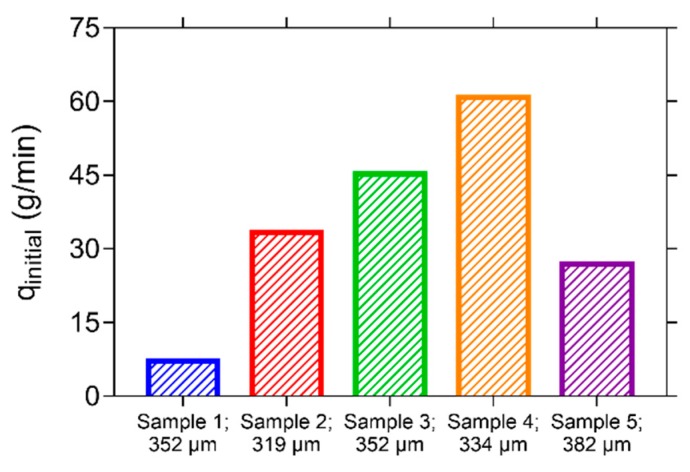
Initial flow rate measured in the water permeability setup for the five different specimens with an indication of the mean crack width.

Figure 15 shows the initial flow rate of the five water permeability specimens, prior to healing. The flow rates vary from 7.1 g/min to a maximum flow rate of 60.9 g/min. The average of these five flow rates is equal to 34.7 g/min with a coefficient of variation (*COV*) equal to 58.0%. This is quite a large variation compared to the crack width which has an average value of 348 µm with a coefficient of variation (*COV*) equal to 6.8% for the five specimens. It has already been noted in previous research that the variation on the flow rate can be an order of magnitude higher than the variation on the crack width [34]. In this previous study the *COV* of the flow rate varied from 9.5% (mean crack width of 307 µm) to 20.5% (mean crack width of 161 µm) for different series. The *COV* of the flow rate in the current study is much higher, which can be explained by the difference in specimen layout and mix composition. In the previous research, prismatic mortar specimens (40 mm × 40 mm × 160 mm) were tested in a permeability setup in which water was induced through one cast-in hole located at 15 mm from the bottom of the specimens. In the current study, the specimens are made from concrete, instead of mortar. The larger aggregate size will make the matrix less homogenous and thus the influence of the internal crack geometry can be very different between specimens. Additionally, in the current study the specimens are much larger (150 mm × 150 mm × 550 mm) and also the water is induced much higher in the specimens (at 92 ± 2 mm from the bottom of the specimens). Therefore, the water has to travel a much larger distance along the crack before it can leak out of the specimens. As a consequence, the influence of the internal crack geometry on the flow rate becomes more pronounced. Furthermore, the reported crack width is measured at surface, whereas the crack width at the location of the glass tubes where the water is introduced into the crack will be different. The influence of these aspects is evident when comparing samples 1 and 3: both have the same crack width at the surface (352 µm), yet the flow rate of sample 3 is six-times larger than the flow rate of sample 1.

**Figure 16:**
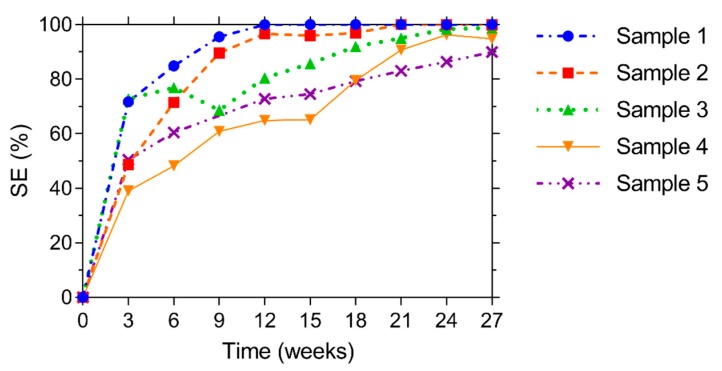
Evolution of the sealing efficiency *SE* of five samples up to 27 weeks of water saturation.

After the initial water permeability test, the specimens were immersed and their flow rate was determined again every 3 weeks, up to a saturation period of 27 weeks. By comparing the flow rate of a sample after a certain healing duration to the initial flow rate of the same sample, the sealing efficiency (*SE*) could be determined according to Equation (2). Figure 16 shows the evolution of *SE* over time for the five different samples. Already after 3 weeks of water saturation, *SE* is at least equal to 39.1%, up to a maximum of 72.8%. After 12 weeks of submersion, the rate of water leakage of sample 1 is almost identical to the evaporation rate. This is also the case for sample 2 after 21 weeks of submersion. After 27 weeks of submersion, *SE* is at least equal to 90% and for three specimens *SE* is even higher than 98.5%, resulting in an average *SE* of 96.7% after 27 weeks of healing in saturation. It can be noticed that there is a slight dip in the curve of sample 3 at a time of 9 weeks. The reason for this is not entirely clear, but could be the result of break age and subsequent washing out of some of the healing product.

Evaluation of the different crack locations for the different specimens after healing showed that not all locations had a complete crack closure. This was also the case for locations on the cracks of sample 1 and 3 which had a negligible leakage. This indicates that the flow path of the water is obstructed in the interior of the cracks rather than near the crack mouth. In other words, sufficient healing products are produced in the interior of the crack to prevent an outflow of water. It is again remarked that the reported mean crack widths of the different specimens in Figure 15 are indications of the crack width near the crack mouth. At the location where the water was introduced in the specimens via the glass tubes, the crack width will have been much smaller, allowing for a more complete crack closure, see also Section 4.2.

## 5. Inspection of the Trial Site

**Figure 17:**
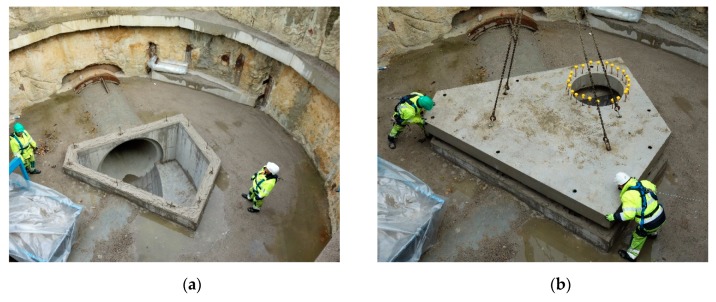
(**a**) Building pit prior to installation of the roof slab. (**b**) Installation of the roof slab on the inspection chamber inside the construction pit.

Approximately 5 weeks after casting, the roof slab was moved from its position next to the construction pit and was installed on the inspection room. Figure 17a shows the building pit prior to installation of the roof slab and Figure 17b shows the installation of the roof slab on the inspection chamber. More than one year after casting, the bottom side of the roof slab was examined. No cracks were found during this inspection. A possible explanation could be that the backfilling of the construction pit happened quite late, therefore, the concrete had obtained its full strength potential when it was loaded by the ground layer. Additionally, it is unlikely that the most severe load combination (including an axis load of a fully loaded truck, i.e., de dominant load) occurred. The backfilling was one of the last tasks on the construction site and so there was almost no more construction traffic.

**Figure 18:**
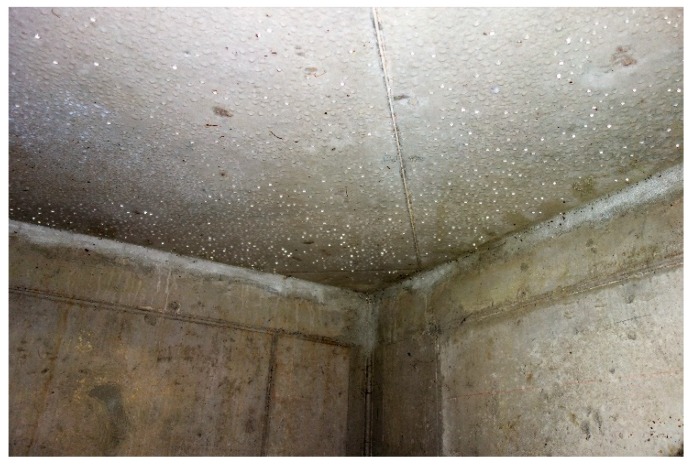
Condensation on the bottom side of the roof slab when the inspection pit and the connecting drainage pipes are operational.

Despite the fact that no cracks were found, it was positive to observe that there was condensation on the bottom side of the roof slab, see Figure 18. About 30% of the roof slab was covered by large condensation drops, even though the water level in the drainage pipes was quite low. This indicates that the conditions in the inspection chamber are favorable in case cracks would form. As indicated in Section 4.2, contact with liquid water is required in order to have a significant crack closure due to healing.

## 6. Conclusions

A bacterial healing agent (MUC^+^) together with nutrients was successfully added to a concrete mix in an industrial concrete mixer. This bacterial concrete was transported to a construction site with a mixing truck and was used to cast a roof slab for an inspection pit as well as accompanying lab specimens. Tests on the lab specimens indicated:

(1) The need for liquid water to start CaCO_3_ production of the bacteria;
(2) The best visual crack closure for specimens subjected to wet-dry conditions, compared to humid conditions (>95% RH) or permanent saturation;
(3) A more consistent visual crack closure (also at higher mean crack widths) for crack locations at the bottom of specimens, compared to cracks on the top or on the side of specimens;
(4) A negligible improvement in capillary water absorption after healing for cracks larger than 300 µm, which can be attributed to an incomplete crack closure at the surface;
(5) A large influence of the crack tortuosity on the initial water permeability of different specimens, when the crack width is nearly constant;
(6) A nearly perfect regain in liquid tightness tested by water permeability (>90%, for three out of five specimens >98.5%), which can be explained by smaller crack widths at the location of the water introduction in the specimens than at the surface of the specimens (>300 µm).
(7) An on-site inspection, more than 1 year after casting, showed no sign of cracking at the bottom of the roof slab. Most likely the design cracking load was not obtained. However, the inspection showed favorable conditions for healing if the slab would crack in the future.

This large scale demonstrator of self-healing concrete has highlighted some important aspects which will be taken into account in future research. Not all elements that can be made out of self-healing concrete are desirable as a demonstrator since they might not be readily accessible for inspection. This project has also highlighted that a manual addition of healing agent into an industrial concrete mixer is not an ideal solution, as the mixing time has to be extended which might result in an increased air content. Additionally, structural designs are done taking into account a wide variety of realistic load cases, yet it is possible that the most severe load combination does not occur in reality (or takes a long time to occur). In any case, this application of self-healing concrete has been an important step in gaining confidence of designers, contractors, as well as engineers at concrete mixing plants to consider self-healing concrete as an option for challenging applications.

## Figures and Tables

**Table 1:** Concrete composition.

Components	Amount
CEM III/B 42.5 N (kg/m³)	430
Aggregate (0−2 mm) (kg/m³)	171
Aggregate (0−4 mm) (kg/m³)	581
Aggregate (2−6 mm) (kg/m³)	162
Aggregate (8−22 mm) (kg/m³)	815
Water/Cement (-)	0.46
Superplasticizer A (m% of Cement)	0.26
Superplasticizer B (m% of Cement)	0.23
Self-Healing Agent MUC^+^ (kg/m³)	4.3
Urea (kg/m³)	4.3
Calcium Nitrate Tetrahydrate (kg/m³)	4.3

**Table 2:** Compressive strength determined on cubes with a side of 150 mm.

Age	Compressive Strength
7 days	30.7 MPa
28 days	45.0 MPa
93 days	54.7 MPa

**Table 3:** Sorption coefficient and crack width of cracked specimens before and after healing, as well as sorption coefficient of uncracked specimens after healing, and sealing efficiency *SE*.

	Before Healing			After Healing			*SE*
	Cracked		Uncracked	Cracked		Uncracked	
	Crack Width (µm)	*SC_crack._* (g/√h)	*SC_uncr._* (g/√h)	Crack Width (µm)	*SC_crack._* (g/√h)	*SC_uncr._* (g/√h)	
1	335	5.239	1.696	53	4.662	1.322	15.3%
2	374	4.844	1.823	156	4.814	1.570	0.9%
3	351	4.832	1.664	184	4.687	1.509	4.3%
Mean	353	4.97	1.73	131	4.72	1.47	6.8%

## References

[1] Cailleux E., Pollet V. Investigations on the development of self-healing properties in protective coatings for concrete and repair mortars. Proceedings of the 2nd International Conference on Self-Healing Materials.

[2] De Maria J., Caprani C., Guo D. (2018). Long span bridges-current age & design life—A global survey. International Conference on Bridge Maintenance, Safety and Management (IABMAS) 2018.

[3] Steel T. (2018). Belgische investeringen in beton lopen hopeloos achter. De Tijd.

[4] Huang L., Krigsvoll G., Johansen F., Liu Y., Zhang X. (2018). Carbon emission of global construction sector. Renew. Sustain. Energy Rev..

[5] De Belie N., Gruyaert E., Al-Tabbaa A., Antonaci P., Baera C., Bajare D., Darquennes A., Davies R., Ferrara L., Jefferson T. (2018). A review of self-healing concrete for damage management of structures. Adv. Mater. Interfaces.

[6] Ferrara L., Van Mullem T., Alonso M.C., Antonaci P., Borg R.P., Cuenca E., Jefferson A., Ng P.-L., Peled A., Roig-Flores M. (2018). Experimental characterization of the self-healing capacity of cement based materials and its effects on the material performance: A state of the art report by COST Action SARCOS WG2. Constr. Build. Mater..

[7] Jefferson T., Javierre E., Freeman B., Zaoui A., Koenders E., Ferrara L. (2018). Research Progress on Numerical Models for Self-Healing Cementitious Materials. Adv. Mater. Interfaces.

[8] Tittelboom K.V., Wang J., Araújo M., Snoeck D., Gruyaert E., Debbaut B., Derluyn H., Cnudde V., Tsangouri E., Van Hemelrijck D. (2016). Comparison of different approaches for self-healing concrete in a large-scale lab test. Constr. Build. Mater..

[9] Tziviloglou E., Van Tittelboom K., Palin D., Wang J., Sierra-Beltrán M.G., Erşan Y.Ç., Mors R., Wiktor V., Jonkers H., Schlangen E. (2016). Bio-Based Self-Healing Concrete: From Research to Field Application. Fortschritte der Hochpolymeren-Forschung.

[10] Araújo M., Chatrabhuti S., Gurdebeke S., Alderete N., Van Tittelboom K., Raquez J.-M., Cnudde V., Van Vlierberghe S., De Belie N., Gruyaert E. (2018). Poly(methyl methacrylate) capsules as an alternative to the “proof-of-concept” glass capsules used in self-healing concrete. Cem. Concr. Compos..

[11] Gruyaert E., Debbaut B., Kaasgaard M., Sorensen H.E., Pelto J., Branco V., Malm F., Grosse C., Price E., Krüger M., Schutter G.D., De Belie N., Janssens A., Bossche N.V.D. (2017). Evaluation of the performance of self-healing concrete at small and large scale under laboratory conditions. 14th International Conference on Durability of Building Materials and Components (XIV DBMC), Ghent, Belgium, 29–31 May 2017.

[12] Keskin S.B., Keskin O.K., Anil O., Şahmaran M., Alyousif A., Lachemi M., Amleh L., Ashour A.F. (2016). Self-healing capability of large-scale engineered cementitious composites beams. Compos. Part B Eng..

[13] Ferrara L., Albertini I., Gettu R., Krelani V., Moscato S., Pirritano F., Flores M.R., Pedro S., Theeda S.M. (2015). Self healing of Cement Based Materials Engineered Through Crystalline Admixtures: Experimental Results from A Multinational University Network. Spec. Publ..

[14] Luo M., Qian C.-X., Li R.-Y. (2015). Factors affecting crack repairing capacity of bacteria-based self-healing concrete. Constr. Build. Mater..

[15] Roig-Flores M., Moscato S., Serna P., Ferrara L. (2015). Self-healing capability of concrete with crystalline admixtures in different environments. Constr. Build. Mater..

[16] Wang J., Dewanckele J., Cnudde V., Van Vlierberghe S., Verstraete W., De Belie N. (2014). X-ray computed tomography proof of bacterial-based self-healing in concrete. Cem. Concr. Compos..

[17] Snoeck D., Van Tittelboom K., Steuperaert S., Dubruel P., De Belie N. (2014). Self-healing cementitious materials by the combination of microfibres and superabsorbent polymers. J. Intell. Mater. Syst. Struct..

[18] Van Tittelboom K., De Belie N. (2013). Self-healing in cementitious materials—A review. Materials.

[19] Dry C. (2001). In-Service Repair of Highway Bridges and Pavements by Internal Time-Release Repair Chemicals.

[20] Beltran M., Jonkers H. (2015). Crack self-healing technology based on bacteria. J. Ceram. Process. Res..

[21] Wiktor V., Jonkers H. (2016). Bacteria-based concrete: From concept to market. Smart Mater. Struct..

[22] Davies R., Teall O., Pilegis M., Kanellopoulos A., Sharma T., Jefferson A., Gardner D., Al-Tabbaa A., Paine K., Lark R.J. (2018). Large scale application of self-healing concrete: Design, construction and testing. Front. Mater..

[23] Teall O., Davies R., Pilegis M., Kanellopoulos A., Sharma T., Paine K., Jefferson A., Lark R., Gardner D., Al-Tabbaa A. Self-healing concrete full-scale site trials. Proceedings of the 11th fib International PhD Symposium in Civil Engineering.

[24] Al-Tabbaa A., Litina C., Giannaros P., Kanellopoulos A., Souza L. (2019). First UK field application and performance of microcapsule-based self-healing concrete. Constr. Build. Mater..

[25] Wiktor V., Jonkers H. (2015). Field performance of bacteria-based repair system: Pilot study in a parking garage. Case Stud. Constr. Mater..

[26] Gruyaert E., Debbaut B., Wang J., Arizo A., Branco V., Alakomi H.-L., Beirao A., De Belie N. Evaluation of the self-healing efficiency of cracks in mortar by bioprecipitation or applications of hydrogels. Proceedings of the HEALCON Conference Self-Healing Concrete for Prolonged Lifetime.

[27] De Belie N., Araújo M., Van Mullem T., Gruyaert E. Demonstration projects with self-healing capsule-based and bacteria-based concrete. Proceedings of the International Conference on Interdisciplinary Approaches for Cement-Based Materials and Structural Concrete (SynerCrete’18).

[28] De Belie N., Van Mullem T., Gruyaert E., Van den Heede P. Self-healing concrete to increase service life of a roof plate for an inspection pit in the Oosterweel link: Healing efficiency and preliminary service life estimation. Proceedings of the 4th International Conference on Service Life Design for Infrastructures (SLD4).

[29] Lantis The Oosterweel Link. https://www.oosterweelverbinding.be/oosterweel-link#.

[30] Bravo da Silva F. (2015). Up-Scaling the Production of Bacteria for Self-Healing Concrete Application. Ph.D. Thesis.

[31] Bravo da Silva F., De Belie N., Boon N., Verstraete W. (2015). Production of non-axenic ureolytic spores for self-healing concrete applications. Constr. Build. Mater..

[32] (2005). CEN Eurocode 2: Design of Concrete Structures-Part 1-1: General Rules and Rules for Buildings.

[33] Feiteira J. (2017). Self-Healing concrete-Encapsulated polymer precursors as healing agents for active cracks. Ph.D. Thesis.

[34] Van Mullem T., Gruyaert E., Debbaut B., Caspeele R., De Belie N. (2019). Novel active crack width control technique to reduce the variation on water permeability results for self-healing concrete. Constr. Build. Mater..

[35] Reinhardt H., Jonkers H., Van Tittelboom K., Snoeck D., De Belie N., De Muynck W., Verstraete W., Wang J., Mechtcherine V. (2013). Recovery against Environmental Action. RILEM State-of-the-Art Reports.

[36] Edvardsen C. (1999). Water permeability and autogenous healing of cracks in concrete. Mater. J..

[37] Van Tittelboom K., Gruyaert E., Rahier H., De Belie N. (2012). Influence of mix composition on the extent of autogenous crack healing by continued hydration or calcium carbonate formation. Constr. Build. Mater..

[38] Neville A. (2002). Autogenous healing—A concrete miracle?. Concr. Int..

[39] Homma D., Mihashi H., Nishiwaki T. (2009). Self-healing capability of fibre reinforced cementitious composites. J. Adv. Concr. Technol..

